# Supplementary material for: Protection Against Pneumonia Induced by Vaccination with Fimbriae Subunits from Klebsiella pneumoniae
Source: Vaccines (Basel). 2025 Mar 11;13(3):303. doi: 10.3390/vaccines13030303 (PMC11945627; doi:10.3390/vaccines13030303)
Supplement: Supplementary file 1 [file vaccines-13-00303-s001.zip › vaccines-3451244-supplementary.pdf]

PROTECTION AGAINST PNEUMONIA INDUCED BY VACCINATION WITH FIMBRIAE SUBUNITS FROM *Klebsiella pneumoniae*

Supplementary Material

1. Clinical strain screening

Different *K. pneumoniae* clinical isolates (Table S1) were compared through *in vitro* agglutination and biofilm formation, for selection of a strain that would be suitable for the challenge experiment. The strains were selected based on their background, infection site and phenotypic characteristics.

Supplementary Table S1: *K. pneumoniae* isolates screened for selection of the challenge studies.

Name	Infection Site	Resistance Characteristics	Genotyping
BM022	Blood culture		KPC+
BM236	Nasal swab		KPC+, OXA-48-, VIM-, NDM-
BM505	Blood culture	MDR Isolate	KPC+
BM567	Blood culture	ESBL+	
BM611	Tracheal secretion/Blood culture	MDR Isolate	KPC+
BM1041	Tracheal secretion	MDR Isolate	KPC+

2. *In vitro* agglutination and biofilm formation assays

The selected clinical isolates were tested for fimbriae expression through an yeast agglutination assay performed as described in [1], and their agglutination ability was defined by weak or strong based on reaction speed and intensity. Supplementary Figure 1A shows the results of this analysis. Among the six *K. pneumoniae* isolates tested, two displayed strong mannose-dependent agglutination upon contact with yeast, BM567 and BM611. This indicates that fimbriae (particularly type 1 fimbriae) are expressed by these strains.

Next, biofilm formation by each strain was measured through an *in vitro* assay in 96 wells microplate. The strains were grown overnight, diluted to an O.D. (600_{nm}) 0.1 in Luria-Bertani broth (without supplementation) and 100µL of the suspension was added to each well of a flat-bottom polystyrene plate. The samples were incubated statically for

24 hours at 37ºC. The supernatant was discarded, the wells, washed and 100µL of 1% crystal violet solution was added to each well. Following another wash, the adhered biofilm was solubilized with 30% acetic acid and the absorbance, measured in the Expert Plus plate reader (Oasys) at a 600 nanometers wavelength. As shown in Figure S1B *K. pneumoniae* BM236, BM567 and BM611 displayed the highest absorbance after crystal violet staining, indicating increased biofilm formation. BM567 and BM611 showed higher biofilm formation when compared to the other strains

Upon compiling the results of the agglutination and biofilm formation assays, the BM567 isolate demonstrated both strong agglutination and robust biofilm formation and was, therefore, selected for this study.

A

Isolate	Agglutination Ability
BM022	Weak
BM236	Weak
BM505	Weak
BM567	Strong
BM611	Strong
BM1041	Weak

B

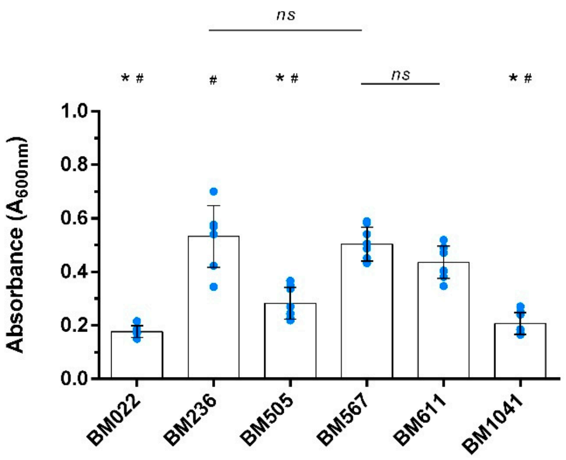

Supplementary Figure S1. *In vitro* screening of the *K. pneumoniae* clinical isolates of their phenotypical characteristics of agglutination (A) and biofilm formation (B). * Indicates statistical difference when compared to BM567 and $^{\#}$ represents significant statistical difference following comparison to BM611. *ns* states that no significative statistical difference was found ($p < 0.05$).

3. *In vitro* bactericidal assay

Resistance of *K. pneumoniae* BM567 to complement-mediated killing was evaluated through an *in vitro* bactericidal assay. The bacteria were cultured in Luria-Bertani (LB) medium until reaching an approximate concentration of 3.5 x 10^8 CFU/mL, then collected by centrifugation (2,000 g for 5 minutes) and washed once with sterile PBS. A 50 µL aliquot of the bacterial suspension was incubated with serum from immunized animals (heat-inactivated) or with the control serum from sham-immunized mice, adjusting the final serum concentration to 5%. Bacterial opsonization was performed for 30 minutes at 37°C. Following opsonization, normal mouse serum was added to a final concentration of 10%, and the final volume was adjusted to 100 µL using opsonophagocytosis buffer (Hank's Balanced Salt Solution), containing bovine gelatin (NaCl 0.14 M, KCl 0.005 M, KH2PO4 0.0003 M, NaH2PO4 0.0004 M, MgSO4·7H2O 0.0004 M, CaCl2·2H2O 0.00125 M, MgCl2·6H2O 0.0005 M, bovine gelatin 0.1%, anhydrous dextrose 1 g/L, NaHCO3 0.35 g/L). The mixture was incubated for an

additional hour at 37°C, and the suspension was subsequently plated onto LB agar for colony-forming unit (CFU) counting. Survival was measured by CFU counting and comparison among groups was performed by ANOVA with a Dunnet posttest. The results are shown in Supplemental Figure 2. Opsonization of the bacteria with sera from vaccinated mice did not affect survival, indicating that this strain is resistant to the bactericidal effects of complement (MAC formation).

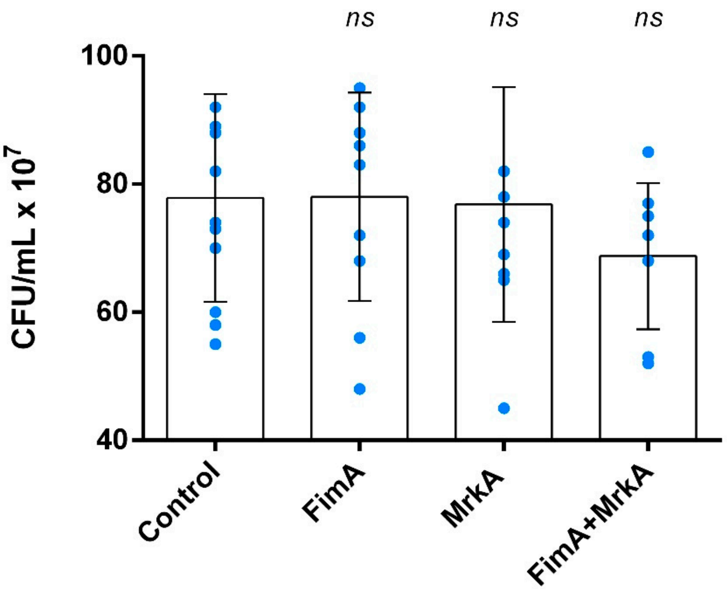

Supplementary Figure S2. Complement mediated killing of *K. pneumoniae BM567*
The bars indicate the number of surviving bacteria after treatment. Comparisono among vaccinated and control sera were performed by ANOVA with a Dunnet posttest. *ns*= non-significant in comparison with control.

4. References

1. Pacheco, T.; Gomes AÉ, I.; Siqueira, N.M.G.; Assoni, L.; Darrieux, M.; Venter, H.; Ferraz, L.F.C. SdiA, a Quorum-Sensing Regulator, Suppresses Fimbriae Expression, Biofilm Formation, and Quorum-Sensing Signaling Molecules Production in Klebsiella pneumoniae. *Front Microbiol* **2021**, *12*, 597735, doi:10.3389/fmicb.2021.597735.
